# Evaluation of a web based tool to improve health behaviours in healthcare staff

**DOI:** 10.1186/1755-7682-7-44

**Published:** 2014-10-08

**Authors:** Hugo C van Woerden, Kathryn Ashton, Christopher Garlick, Andrew Hurley, Andrew Cooper, Alan Willson, Ray Henry, Vasiliki Kiparoglou, Christopher Potter

**Affiliations:** Institute of Primary Care & Public Health, Cardiff University School of Medicine, Neuadd Meirionnydd, Heath Park, Cardiff, CF14 4YS UK; Public Health Wales, Haydn Ellis Building, Maindy Road, Cardiff, CF24 4HQ UK; 53 Pinecrest Drive, Cardiff, CF14 9DW UK; Public Health Wales, 14 Cathedral Road, Cardiff, CF11 9LJ UK; Public Health Wales, Temple of Peace and Health, Cathays Park, Cardiff, CF10 3NW UK; Oxford NIHR Biomedical Research Centre, Joint Research Office, Block 60, Churchill Hospital Old Rd, Oxford, OX3 7LE UK

## Abstract

**Background:**

A web-based tool was developed and piloted by being made available to healthcare staff in Wales from September 2012 to March 2013. This evaluation included two primary outcome measures: general health and mental well-being, and six secondary outcome measures: sickness absence, alcohol use, healthy eating, smoking, physical activity and maintaining a healthy BMI. The aim was to assess the feasibility of a web-based tool to improve health behaviours in healthcare staff.

**Methods:**

Healthcare staff joined via a website, chose two of five challenges, and recorded their health behaviours using an online tool on a regular basis. Evaluation was undertaken by comparing baseline and follow up questionnaires.

**Results:**

1708 individuals explored the programme’s website, of whom 1320 selected two lifestyle challenges to address. Of these 346 individuals (26.2%; 346/1320) completed the end of project evaluation questions for the main outcome and provided the basis of the evaluation. Comparing pre:post data among respondents who engaged with the programme as a whole, self-reported general health status improved in 35.3% (n = 122, p = 0.001); mental health status improved in 33% (n = 110, p = 0.02); alcohol consumption score (AUDIT-C classification) fell in 27.2% (n = 71, p = 0.001); reported fruit and vegetable consumption (7 day recall) increased (p = 0.001); average time spent on vigorous exercise increased from 40.6 minutes a week to 67.6 minutes a week (p = 0.001); and 41 individuals noted a positive change to their BMI classification category (p = 0.001).

**Conclusions:**

Combining interactive web-based tools as part of a multi-media programme is feasible, increases health behaviours and generates interest among a proportion of the healthcare workforce. Further work is required to improve maintenance of engagement over time.

## Background

There is increasing evidence that work-based health promotion interventions may be an effective, low cost method for improving health outcomes [1]. Interactive, personalised, multimedia behaviour change programs are increasingly being utilised to this end [2]. Previous studies have indicated that such health promotion initiatives may be more effective and more engaging for users than alternative formats such as paper-based information [3–5], and may also be more effective at empowering clients [6]. In previous research, internet-based health promotion programmes typically have had a small but significant effect on health behaviour [7]. Effect sizes are typically larger for programmes that have a strong theoretical basis, incorporate more behaviour change techniques, and involve additional motivational communication with users such as SMS messages or emails [7]. Given the potential reach and cost-effectiveness of internet based health promotion interventions the approach merits further development.

This real world evaluation grew out of a desire to develop a web-based platform with modules on healthy eating, physical activity, maintaining a healthy weight, moderate alcohol intake, and smoking cessation. The project recognised the importance of drawing on the following theoretical models. The health belief model [8], which is based on changing perceptions of health problems, addressing perceived barriers and emphasising the perceived benefits of behaviour change. The theory of planned behaviour [9], which is based on informing and raising awareness to change attitudes, increasing motivation and enabling behaviour change. The self-regulatory model [10], which is based on supporting individuals in taking an active role in managing their own condition. The PDSA (plan, do, study, act) model [11], which includes setting specific manageable goals, reflecting on their progress, and adjusting behaviour [12].

The aim of the study was to assess the feasibility of a web-based tool to improve health behaviours in healthcare staff. The primary outcomes assessed were self-reported general health and mental well-being. Six secondary outcome measures were recorded: sickness absence, alcohol use, healthy eating, smoking, physical activity, and achieving a healthy BMI. The final aim was to assess reasons for early drop out in participants. Changes were assessed over a six month period. The study was limited to healthcare workers in a range of organisations in Wales.

## Methods

A web-based platform, based on the theoretical models referred to above, was designed, developed and piloted. A communication campaign was developed which promoted the programme using publicity material, celebrity endorsement, launch events and local champions; details of which are provided elsewhere [13]. Each healthcare organisation in Wales was allocated a number of places for employees to sign-up to the programme based on their relative size. Places within organisations were allocated on a first-come-first-served basis. As these places were rapidly filled, exceeding the initial target of 1,000 participants, additional places were provided to organisations with the highest demand. The tool was provided to healthcare workers rather than patients, as it was hoped that staff would be able to use their daily contact with patients to role model and cascade out positive health behaviours.

Healthcare staff registered with the website, provided baseline information and completed a lifestyle assessment against five health behaviours. This health assessment was designed to facilitate choice of two modules to be undertaken during the study period. The individual modules were designed to provide health information, signpost to resources and services, and provided an opportunity for individuals to record and track their progress against a target over a six-month period.

The intervention encouraged participants to undertake online brief intervention training on a range of lifestyle topics. This was designed to encourage participants to pass on what they had learned to others around them, including patients. Participants were regularly contacted via emails, text messages, newsletters and on Twitter to remind them to access the website and to motivate them to continue with the challenges they had chosen. A web forum was provided where participants could raise or discuss issues. The core of the intervention was the recording of personal data on a weekly basis, which was used to provide personal feedback via a visual display which summarised performance against nationally recommended targets.

At the end of the six month challenge period, participants were directed to an evaluation questionnaire via email, text messages and a link on the front of the website. The evaluation page re-asked the baseline assessment module questions and included questions related to the other primary outcomes (general health and mental health). Information on sickness absence over the preceding six months was requested and an open question was used to gather qualitative information on users’ experience of the programme. The free text responses were reviewed and illustrative responses extracted.

Pre:post intervention change was assessed using baseline and six month data. The outcome variables used for analysis were: the Office for National Statistics ‘harmonised general health question’ assessed on a five point scale from 1 – excellent to 5 – poor [14]; a standard measure of anxiety and depression ( the Patient Health Questionnaire PHQ-4) [15, 16]; a question on ‘the number of days of sickness absence’ and ‘spells of sickness absence lasting over one week’ during the last six months, based on the items included in the Health and Safety Executive’s stress tool and the Well-being in Work workforce survey [17]; the Alcohol Use Disorders Identification Test (AUDIT-C) [18]; an eight-item Food Frequency Questionnaire (FFQ) for consumption of fruit and vegetables [19]; the Fagerström Test assessing nicotine dependence in smokers [20]; the seven-item self-completed version of the International Physical Activity Questionnaires (IPAQ) [21]; and height and weight which was used to calculate BMI. All outcome measures were self-reported.

Those who entered data for six or more weeks were defined as the ‘active’ group for analytical purposes. Anonymised data was extracted and entered into SPSS® version 20 for analysis. Outcome measures were plotted to assess whether they were normally distributed. The paired t-test was used when outcomes were close to a normal distribution and non-parametric tests were used when this was not the case. A statistical test was undertaken in relation to the before and after paired data for each of the predefined outcomes.

A proportion of participants explored the prototype website before it was completed, but did not go on to select a lifestyle challenge on the website. To examine the reasons for this a short web-based questionnaire was sent to the email address of those ‘early dropouts’ to assess why participants had dropped out of the programme at this early stage.

Formal ethical approval was not sought for this project, as this was a developmental evaluation of a service project. However, advice was sought from the Research and Development Office in Public Health Wales and the project was undertaken in full compliance with the ‘Helsinki Declaration’ as laid out in the ‘Handbook of the World Medical Association Policies’.

## Results

A total of 1708 individuals explored the prototype website for the programme before it was completed. Once the programme was up and running 1320 of these individuals registered and selected two lifestyle challenges to address. Response rates for the primary outcomes (those completing the relevant end of project evaluation questions) were: general health question 26.2% (346/1320), and mental health questions 25.2% (333/1320). At least 247 individuals provided baseline and end of module data for one or more lifestyles module questions, allowing for pre:post intervention comparison. The number of respondents providing pre and post data varied slightly across the lifestyle assessment modules. The demographic composition and baseline characteristics of participants are provided in Table 1.

**Table 1:** **Characteristics of individuals who signed up to the programme (n = 1320)**

	Number	Percent
**Gender**		
Male	265	20.2
Female	1048	79.8
**Age (years)**		
16-25	61	4.6
26-35	269	20.4
36-45	409	31.0
46-55	464	35.2
56+	115	8.7
**Socio-economic grouping**		
Managerial, administrative and professional occupations	1261	95.5
**Ethnicity**		
White	1235	93.6
**Self-reported general health**		
Excellent	118	9.0
Very good	448	34.3
Good	559	42.8
Fair	169	12.9
Poor	13	1.0
**Mental Health (PHQ-4)**		
Greater than or equal to 6	85	6.6
**Spells of sickness lasting a week or more in last 6 months**		
One or more	295	22.9
**Alcohol consumption (AUDIT C)**		
Sensible drinking	691	52.3
Hazardous drinking	436	33.0
Harmful drinking	193	14.6
**Body Mass Index (BMI)**		
Underweight/healthy weight	381	28.9
Overweight	470	35.7
Obese	466	35.4
**Smoking**		
Smoked in last 4 weeks	107	8.1
**Exercise**		
More than 150 minutes exercise in last 7 days	152	11.5
**Healthy eating**		
More than 35 portions of fruit and vegetables in last 7 days	606	45.9

The challenge of taking regular exercise was the most popular, selected by 79.6% (1051/1320), while the stop smoking challenge was the least popular, selected by 3.1% (41/1320). The most popular combination of challenges was taking regular exercise and working towards a healthy BMI (52.5%, 693/1320) followed by taking regular exercise and eating healthily (18.6%, 245/1320).

### Baseline data

On initial registration, 8.6% (147/1708) self-reported their health as excellent, 33.8% (577/1708) very good, 41.3% (706/1708) good, 13.4% (229/1708) fair, and 1.1% (19/1708) poor. On the PHQ-4 Anxiety Depression Measure, 6.8% (111/1635) of respondents scored six or more on the 12 point scale (the higher the score, the more likelihood there is of an underlying depressive or anxiety disorder). Overall, 24.5% (404/1652) of individuals reported having one or more spells of sickness lasting for more than a week over the preceding six months. Among the respondents, 40.9% (684/1672) scored their performance at work as 8 out of 10 or better (1 being poor, 10 being excellent) over the 30 days preceding their completion of the Champions for Health assessment questions (Mean 7.8, SD 1.2). A summary of the factors that motivated them to join is provided in Table 2.

**Figure 1:**
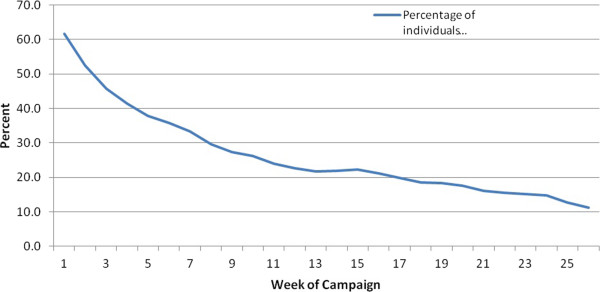
**Decay curve illustrating the percentage of individuals who entered challenge data on a weekly basis.**

Of those individuals who selected two challenges, 64.2% (847/1320) entered weekly challenge data at some point during the programme and 36.7% (484/1320) entered challenge data for six or more weeks (the ‘active’ group). In total, 14.7% (194/1320) of the fully registered individuals entered challenge data for over 20 weeks of the programme. The number of staff continuing to engage with the programme as time progressed in shown in Figure 1.

**Table 2:** **factors that motivated joining the programme (n = 349)**

Why did you decide to take part?*	Number	Percent
To improve my general health	269	77.1
To get fitter	207	59.3
To feel better about myself	145	41.5
To improve my body image	124	35.5
To have more energy	122	35.0
To prevent ill health later in life	118	33.8
To improve my mental well-being	78	22.3
To help me encourage others to improve their health	68	19.5
My local Champions for Health link person encouraged me to take part	24	6.9
I was inspired by the celebrities supporting the programme	4	1.1
I was inspired by the case studies I read about other people taking part	5	1.4

*Note: individuals could select more than one answer.

Throughout the programme, 192 individuals responded to motivational text messages which were sent as part of the communications strategy. Of these individuals, 139 responded to text messages on more than one occasion. As expected, a higher percentage of those who responded to text messages were in the ‘active’ group than those who didn’t respond to any of the text messages (48% and 34.9% respectively).

### Pre:post intervention changes in lifestyle

Of those who responded to the evaluation questionnaire, 72.8% (254/349) were in the ‘active’ group, and had entered weekly data for at least six weeks. Conversely, 10.6% (37/349) of respondents to the evaluation questionnaire presented at the conclusion of the programme were individuals who had not entered challenge data at any point during the six months of the programme.

There was a statistically significant improvement in ‘general health’ in the 346 individuals who recorded a value before and after the programme (Wilcoxon Signed-Rank Test p = 0.001), with a median incremental increase of one point on the 1–5 scale, from 3 (‘good’) to 2 (‘very good’). Overall, 35.3% (122/346) reported a positive change in health status at the end of the programme, with 12.1% (42/346) reporting a negative change in their health status. The remaining respondents reported no change.

The programme had a statistically significant impact on mental health scores in those who recorded values before and after the programme (Wilcoxon Signed-Rank Test p = 0.024) with a median decrease of one point on the PHQ4 scale, from an initial median of one to a median of zero. In total, 33.0% (110/333) of individuals had a positive change mental health after the 6 month period, with 23.4% (78/333) reporting a negative change, 145 respondents reported no change and the remainder did not answer that question.

Gender related difference were relatively small. There were no statistically significant differences between males and females in the proportion experiencing improvement as a result of participating in the programme for General Health (p = 0.59), Mental Health (p = 0.51), alcohol risk score (p = 0.25), weight loss (p = 0.12), fruit and vegetable intake (p = 0.051), or exercise level (p = 0.46).

There was a fall in the average number of self-reported days off sick at the end of the study (3.73 days to 3.63 days) and a fall in the mean number of spells of sickness absence (0.36 to 0.29 days), although this was not statistically significant (Wilcoxon Signed-Rank Test p = 0.154 and p = 0.179 respectively).

Of those who chose to complete the alcohol challenge and recorded baseline and end of study data, the number of ‘sensible’ drinkers increased (8.8% to 42.1%) and the number of ‘harmful’ drinkers decreased (35.1% to 8.8%). Statistically significant differences were present in BMI in those who recorded values before and after the programme (Wilcoxon Signed-Rank Test p = 0.001). A total of 41 people recorded a positive change to their BMI classification category. The proportion of respondents in the ‘Obese’ BMI category decreased from 33.6% (88/262) to 26.3% (69/262), and the total in the ‘Healthy Weight’ category increased from 29.4% (77/262) to 34.7% (91/262). Overall, 157 individuals who responded to the evaluation lost weight over the 6 month programme, with the highest weight loss being recorded at 22 kg (3st 7 lb). In total 749 kg (118st 1 lb) were reported to have been lost over the whole study period, with a mean weight loss of 4 kg (10.5 lb) per individual who reported weight loss. A proportion (76 individuals) reported putting on weight with a mean increase of 1.5 kg (3.4 lb).

The number of fruit and vegetables consumed over the last seven days increased in those who recorded values before and after the programme (paired t-test p = 0.001). The mean number of portions eaten over a 7-day period had increased from 49 portions to 59 portions.

There was no significant difference in the total time spent on all types of exercise over the past 7 days in those who recorded values before and after the programme (paired sample t-test p = 0.388). *Post hoc* analysis indicated a significant increase in the amount of time spent on vigorous exercise in those who selected the physical activity challenge (increase in mean time from 40.6 minutes a week to 67.6 minutes a week (paired sample t-test p = 0.001).

The impact of the intervention on levels of smoking could not be assessed as few participants responded to the pre and post intervention questions on smoking (n = 9).

There were no major differences to the health outcomes whether individuals were members of the ‘active’ group (entering six or more weeks of challenge data on the website) or the ‘non-active’ group.

### Views on the programme

A range of questions were included to assess the communication aspects of the programme. Based on the Stages of Change Model [22], the majority of respondents undertaking final evaluation indicated that they had already decided that they wanted change and were unsure about how to put it into practice or had previously failed, or had already started and wanted the help to maintain (80.7%, 276/342). Only two people stated that they were at the contemplation stage of the cycle when they first took part.

Some participants (19.5%, 68/349) stated that they took part to help others to improve their health. Only 1.1% (4/349) stated that they were inspired by the celebrities who had endorsed the programme.

When questioned, 87.4% (305/349) of respondents to the evaluation questionnaire stated that they picked the challenges which they ‘thought would make the most difference to their health’. A total of 16.9% (59/349) chose the challenges which they thought would be most achievable.

Table 3 summarises whether respondents would pass on what they had learned and the extent to which the programme helped different aspects of behaviour. Respondents could select more than one option. Participants seemed willing to pass health messages to family, friends and colleagues but less willing to pass these messages on to the patients they treat. Table 4 indicates that over 90% of participants who responded believed that the programme had helped make changes to their own health behaviour.

**Table 3:** **Whether respondents would pass on what they learnt (n = 349)**

Would you pass on what you have learnt to:	Frequency	Percent
Family and friends	235	67.3
Colleagues	158	45.3
Patient/Service users	50	14.3
Members of the public	31	8.9
None of the above	68	19.5

**Table 4:** **Respondents views as to whether the programme has helped make changes to their own health behaviour**

	Frequency	Percent
**Make changes to own health behaviour**		
Extremely	14	4.1
Very much so	93	27.1
Somewhat	117	34.1
A little	91	26.5
Not at all	28	8.2

There was a mixed response to the usefulness of the communication channels used throughout the programme. The most useful form of communication was deemed to be via email (79.4%, 277/349), with text messages the second most useful (62.4%, 218/349). However, a few individuals found the text messages “*intrusive”*. A number of individuals stated that they did not have access to Twitter in work, and many stated that they didn’t have access to it at home either. Several suggested using Facebook as a channel of communication. Another suggestion was the development of a smart phone App on which to access the platform.

In total, 84.2% (294/349) said that they would continue with changes to their lifestyle after the programme had ended.

Overall, 28.1% (98/349) of respondents to the evaluation questionnaire stated that they would recommend the programme to a colleague and 27.5% (96/349) would ‘probably’ recommend to a colleague. Participants were asked to expand on their views in a free text box. A sample of the comments that were made is provided in Table 5. The weekly data entry and process charts which allowed respondents to track their progress by challenge over the 6 month period was most commonly commented on positively. A number of individuals found the website to be “*supportive/motivational when things got tough”*. Some stated that completing the regular challenge data “*acted as a conscience for the number of units [of alcohol] you consume”* and provided an *“incentive”* to keep going. The idea of enhancing motivation is typified by responses such as: *“I think actually recording what you have previously achieved inspires you to continue when things are a little difficult”, “It motivated me at low times”, “Guilty reminder to keep at it”, “felt like someone cared about me doing this when not much support at home and colleagues had given up”.*

**Table 5:** **Additional aspects of the programme which respondents commented on and suggestions for change**

Main themes presented	Key quotes
**Additional aspects of the programme which respondents found helpful**	
Weekly data entry and process charts	*“I think actually recording what you have previously achieved inspires you to continue when things are a little difficult”*
	*“Recording what I did each week and seeing the patterns - I was able to target areas for improvement”*
	*“The main thing which helped me was to be able to log my daily results. This was the single biggest help form the Champions for Health resources”*
Promotion of health in the workforce and endorsement by leaders	*“I’ve worked for the NHS for nearly 30 years and this is the first time I’ve ever seen or been involved in something like this”*
	*“The fact that the NHS is focused on focusing on the health and well being of our workforce was really positive message”*
Increased awareness of lifestyle choices	*“Before C4H I thought I was a pretty healthy person. Doing the assessments shocked me as I didn’t come out as well as I thought so it’s given me a wake-up call”*
Communication	*“I really liked the different routes of communication”*
	*“the text messages did spur me on to be more healthy and have since joined local slimming club and am doing really well”*
**Suggestions for change**	
Website	*“make the website more user friendly and easier to input your details”*
	*“the website was not easy to navigate- this put me off using it”*
	*“the layout of the dashboard and website- quite confusing”*
	*“Maybe just a little booklet to help me find my way around the website”*
Communications directed towards making and sustaining change	*“There was no understanding of the process of making and sustaining change”*
Media awareness	*“More national media coverage would have been useful- with stories in local newspapers to keep profiles raised in communities in which these people live”*
Channels of communication	*“Have an app for entering information, Don’t make it so arduous-filling it in everyday was too much”*
	*“maybe a phone app as this would have likely led to me continuing to monitor my progress with you”*
Tailoring communication channels and level of engagement	*“I didn’t realise beforehand I would be receiving texts messages- I would have preferred not to receive these”*
	*“Perhaps more checks along the way to help the quiters come back”*
	*“Would there be a way of switching off the texts and emails should circumstances be that you no longer wanted them or perhaps reduce the frequency”*
Proactive events to promote interaction and contact with other Champions	*“When people sign-up – they should be told that their names will be added to a list for viewing by the C4H team unless they specifically request anonymity”*
	*“It would be helpful to know who the other Champions were”*
	*“Maybe some proactive engagement would have helped – e.g. events (maybe a walk/run- seminars-drop in advice/screening day)”*
	*“Having a more interactive link to smoking cessation programmes- virtual support groups/chat rooms/buddying systems with people who you don’t know”*
	*“More strongly encouraging a buddy system. As I work on the nurse bank I don’t have regular colleagues to support me (or to support)”*
Encouragement at the local level	*“I did not have the time or inclination to keep going online- a local contact and motivator would be ideal to keep you focused”*
	*“I need someone to check up on me”*
	*“A little disappointed that there was limited encouragement from Health Board level to take part”*
	*“I think the recording of weight should be done at work by a nominated person”*
	*“I approached the link person for help with losing weight – they said they’d call me to discuss but they never did”*
Seasonality	*“The Winter period is not the time to eat salad and try cycling/power walking in freezing conditions! Overall the concept was good- just bad timing”*
Move from top-down to bottom-up approach	*“Find out what staff want and what might help them”*

Respondents also found support from their family and friends particularly helpful, for example, *“my daughter was the best supporter as we went to the gym together”.*

### Survey of ‘early dropouts’

As already stated, a total of 1708 individuals explored the prototype website for the programme before it was completed, but 22.7% (388/1708) did not go on to select or lifestyle challenges on the website. To examine the reasons for this a separate web-based questionnaire was sent to the email address of those who had explored the prototype website but not participated further. This survey was designed to assess why participants had dropped out of the programme at this early stage. Of these, 12.1% (47/388) responded. The main reasons identified from free text for not continuing were: technical or functional difficulties which had prevented them or distracted them from participating in the programme (n = 14), leaving or changing their post around the time when the programme process was beginning (n = 4), continuing lifestyle improvements outside of the scheme (n = 3), personal reasons (n = 3), forgotten/never started (n = 2). Since initially registering with the programme, 80.9% (38/47) stated that they had taken other steps to improve their health. The comments given indicate that part of the trigger for action was inspiration from this programme: *“a colleague and I used all the principles, we just did it between us…so I would still say it was a success, thanks v. much”, “I quit smoking on Sept 1*^*st*^*2012, It’s something that I am so very proud of. Champions for Health and the Smoking ban within all Hospital grounds inspired me to quit”.*

## Discussion

This study has built on a range of theoretical approaches, combining interactive web-based tools with regular communication, as this approach has been shown to improve health behaviours in previous studies. It has applied these concepts to healthcare staff and strengthened the evidence base that interventions of this nature are valuable as public health tools. This study was designed as a real world evaluation of effectiveness in day to day practice. Although the use of such pragmatic studies has provoked some controversy, there is a place for real world evaluations of health improvement tools [23].

Only 21% of those who registered with the programme undertook both the baseline and follow-up evaluation questionnaires. In part, this may have been because the questionnaire used to evaluate the programme may have been seen as distinct from data participants entered into the website for their own benefit. The drop-out rate in this study was typical for projects of this nature [24, 25], but is still much higher than one would wish (Figure 1). Methods to improve retention of participants, , need to be incorporated and evaluated to address this issue, perhaps building on ‘game mechanics’ or other reward systems that have been utilised in computer games.

A high percentage of participants were female, but this was in relatively close approximation to the proportion of the workforce that are female in NHS Wales [26]. The scheme primarily captured office-based staff and the number of participants in the technical and craft occupations was relatively small. Web based programmes by definition require participants to be able to access a computer. This means that the study inadvertently selected those in a specific subset of socio-economic groups (professionals, managers, and administrators) who could access a computer easily as part of their work schedule, compared to those without access to a work computer e.g. porters, kitchen orderlies, general drivers etc. This could partly be addressed by making such resources available on hand held devices such as smart phones.

The percentage of all participants who smoked (8.5%) was much lower than the proportion in the Welsh population overall as determined by the Welsh Health Survey [27], which estimated that 23% of the Welsh population smoke, and the General Household Survey [28], which estimated that 20% of the adult population of Great Britain smoke. Smoking rates may also have been under-reported, particularly as this was the lifestyle assessment topic with most missing data (19.7%). The low number of smokers among participants may also be related to the socio-economic status of the sample and to a greater awareness of the harms associated with smoking in healthcare staff.

In this study, only seven participants, representing 0.5% of those where BMI was known, were underweight. Separate analysis of this group was therefore not possible. However, in general, it is worth undertaking separate analysis of those who are underweight, as this group often has distinct behavioural characteristics.

Although there are some issues around the specificity of AUDIT-C, which can be low in some populations [18], the AUDIT-C has been shown to be an effective screening tool for problem drinking [29] and is widely used in public health practice.

The short eight-item FFQ that we used was developed and validated in the Netherlands [19]. However, the specific items included in FFQs need to be culturally appropriate and easy to complete. We therefore adapted the FFQ categories in this questionnaire, based on the British Heart Foundation categorisation of fruit and vegetable portions included in their guide to maintaining healthy weight [30].

This study measured pre:post intervention change and recorded free text comments that can be used to develop and refine the service for the future. However, it was not an RCT and in the absence of a control group the true effect size cannot be determined. The cost-effectiveness of the initiative has also not been calculated. Once established, it is estimated that the cost per user would only be a few pounds per year. All health data was self-reported by individuals and may be subject to recall, reporting and response bias as no direct measurements were made to verify self-reported activity or health status.

The study may also have been vulnerable to the effects of seasonal variation. Pre-programme assessment took place in September, the study period was October to March and post-programme assessment took place in April.

## Conclusions

The project has demonstrated that this approach to health improvement is feasible and generates interest among a proportion of the healthcare workforce in Wales. However, further work is required to improve maintenance of engagement over time. Improvements were recorded across self-reported health and lifestyle variables in the cohort of individuals who did remain engaged with the programme. Positive associations included self-reported general health (p = 0.001), mental health (p = 0.024), alcohol consumption (p = 0.001), BMI (p = 0.001), healthy eating (p = 0.001) and vigorous levels of physical activity (p = 0.001). The communications strategy employed in the programme also appears to have created widespread awareness of the campaign amongst staff [13]. The information from this evaluation will be useful in continuing to refine this tool further, particularly the proposal that these approaches to health improvement need to be accessible on smart phones.
